# The mediating role of smartphone addiction in the relationship between emotion regulation difficulties and juvenile delinquency

**DOI:** 10.3389/fpsyt.2025.1695691

**Published:** 2025-12-08

**Authors:** Rula O. Alsawalqa, Haroon Abdel Rahim AL- Zawahreh

**Affiliations:** Department of Sociology, The University of Jordan, Aljubeiha, Jordan

**Keywords:** smartphone addiction, emotion regulation, juvenile, delinquency, Jordan

## Abstract

Research has demonstrated the significant roles that smartphone addiction and emotion regulation difficulties play in juvenile delinquency. However, the mediating role of smartphone addiction in the relationship between emotion regulation difficulties and delinquent behavior has not been extensively explored. To address this gap, data were collected via a survey administered to 210 sentenced male juvenile delinquents, aged 14 to 17, who frequently use multiple social media applications via smartphones. Our findings reveal that male juveniles exhibit high levels of smartphone addiction, which significantly disrupts their daily functioning and fosters a strong orientation toward virtual life. These juveniles frequently experience withdrawal symptoms when not using their smartphones. Additionally, they report substantial difficulties in regulating their emotions, including problems with impulse control, non-acceptance of emotional responses, and lack of emotional clarity. These emotional regulation challenges are closely linked to a higher frequency of delinquent behaviors. Furthermore, a significant positive relationship was found between smartphone addiction (which disrupts adaptive functioning) and delinquency. The results further suggest that smartphone addiction mediates the relationship between emotion regulation difficulties and delinquency. Specifically, difficulties in emotion regulation increase the likelihood of smartphone addiction, which in turn escalates the risk of engaging in delinquent behavior. Furthermore, emotion regulation difficulties directly influence juvenile delinquency, independent of smartphone addiction.

## Background

Juvenile delinquency represents a complex social phenomenon that extends beyond legal infractions, impacting the offenders, their families, and society at large. Sociologically, juvenile delinquency reflects patterns of deviant behavior shaped by the interplay of individual vulnerabilities and environmental factors, including weak social control, peer influence, family disintegration, and socioeconomic conditions (1). Adolescents aged 15 to 17 are particularly susceptible to these risk factors due to their critical developmental stage, making them vulnerable to poor parenting, family conflict, exposure to violence, social isolation, and substance use, particularly alcohol and marijuana, which are predictive of juvenile justice involvement (1–3). The broader social environment, including residential context and prevailing social norms, further shapes the likelihood of engaging in delinquent behaviors. These external stressors interact with personal challenges, such as difficulties in emotion regulation, increasing the risk of antisocial conduct (1).

Emotion regulation, defined as the ability to manage which emotions one experiences, when they occur, and how they are expressed (4), has emerged as a key psychological risk factor for juvenile delinquency. Deficits in emotion regulation are associated with mental health disorders, including anxiety, depression, conduct disorder, and ADHD (5). Adolescents who struggle to regulate their emotions may exhibit poor judgment and engage in risky behaviors, including illegal acts (6). Heightened anger and impulsivity often emerge from frustrations linked to emotional dysregulation, leading to aggressive behaviors and increased likelihood of arrest (7, 8). Delinquent youth also experience broader emotional variability, trauma, and maladaptive aggression compared to their non-delinquent peers (9, 10). Psychological resources, such as self-compassion, have been shown to mitigate some of these adverse emotional outcomes (11).

Peer influence and digital environments significantly shape adolescents’ behaviors. Peer groups provide powerful socialization platforms that can encourage both prosocial and deviant actions (12–14). Likewise, social media and smartphone use expose adolescents to peer pressure, risky content, and negative social cues, potentially amplifying delinquent tendencies (15).

Recent literature identifies **smartphone addiction** as a multidimensional construct with complex effects on emotional regulation and behavior. Withdrawal and inefficiency exacerbate negative emotions, whereas escapism and temporary loss of control may provide short-term relief (16). Excessive smartphone use can disrupt family and social bonds, impair adaptive coping, and create cycles of immediate gratification followed by emotional dysregulation (17, 18). Adolescents may rely on smartphones to escape stress, loneliness, or anxiety, reinforcing maladaptive coping strategies and increasing vulnerability to delinquent behavior (19, 20). Evidence indicates that smartphone addiction may have more detrimental effects on adolescent mental health than internet gaming disorder, highlighting the urgency of understanding its links with emotional regulation and problem behaviors (21).

Furthermore, smartphone addiction (commonly known as problematic smartphone use) (22) is linked to psychological, emotional, and cognitive changes that affect juveniles, which requires the attention of professionals in the fields of health and education (23). In the same context, Jeon (24) and Lim (25) emphasized the existence of a mediating relationship between smartphone dependence, emotional problems, and negative upbringing in explaining juvenile delinquency. Notably, certain smartphone applications have been shown to pose real risks to adolescents. The “Blue Whale” game has been linked to harmful behaviors, including self-harm and endangerment of others. In Jordan, a group of students in a southern governorate experienced serious consequences after downloading and participating in the game via social media on their smartphones (15).

In Jordan, the rising juvenile offenses highlight the urgent need to study these phenomena within the local context. According to the 2024 Criminal Statistical Report by the Jordanian Criminal Information Department, juveniles in Jordan committed a total of 2,466 crimes, representing an increase compared to 2,159 offenses recorded in 2023. The distribution of these offenses in 2024 was as follows: 73.36% were crimes against property, 8.84% crimes against public morals and ethics, 8.03% felonies and misdemeanors against persons, 6.16% crimes against public administration, 2.72% crimes against public safety, 0.49% crimes against public confidence, and 0.41% classified as other crimes (26). Most offenders were in their late teenage years (15–17 years old), residing in urban areas, coming from low-income families with limited parental education, with a substantial proportion out of school and a majority of mothers not employed (27, 28). Consistent with these demographic trends, Al-Zawahreh (27) found that emotion regulation difficulties and low self-control were among the strongest psychological predictors of delinquent behavior among Jordanian male juveniles. The study further revealed that excessive smartphone use significantly mediated the relationship between emotional dysregulation and delinquency, indicating that digital engagement may exacerbate maladaptive coping and antisocial tendencies. These findings underscore the relevance of integrating emotional and digital factors in understanding juvenile offending within the Jordanian context.

Many engage in theft-related crimes and exhibit low self-control, illustrating the interplay between environmental pressures, psychological vulnerabilities, and delinquent behavior (23). Problematic digital engagement has also been linked to maladaptive outcomes beyond delinquency, such as disordered eating and impaired self-regulation, underscoring broader behavioral risks (29). Real-world cases, such as the “Blue Whale” game, exemplify how smartphone applications may expose adolescents to harmful behaviors (15).

This study is grounded in General Strain Theory (GST) and Self-Regulation Theory, providing a theoretical framework to examine the interplay between emotion regulation difficulties, smartphone addiction, and juvenile delinquency. GST posits that individuals exposed to strain—defined as experiences or conditions perceived as negative or undesirable—may develop negative emotions, such as anger, frustration, or depression, which can lead to maladaptive coping behaviors, including delinquency (30). Strains that are unjust, severe, or accompanied by low social control particularly increase the likelihood of deviance (31, 32). Complementarily, Self-Regulation Theory emphasizes individuals’ capacity to guide thoughts, emotions, and behaviors toward goal-directed outcomes through self-monitoring and self-control (33, 34). Variations in self-regulatory capacity determine whether adolescents cope adaptively or engage in maladaptive behaviors such as excessive smartphone use or delinquent acts.

**Figure 1:**
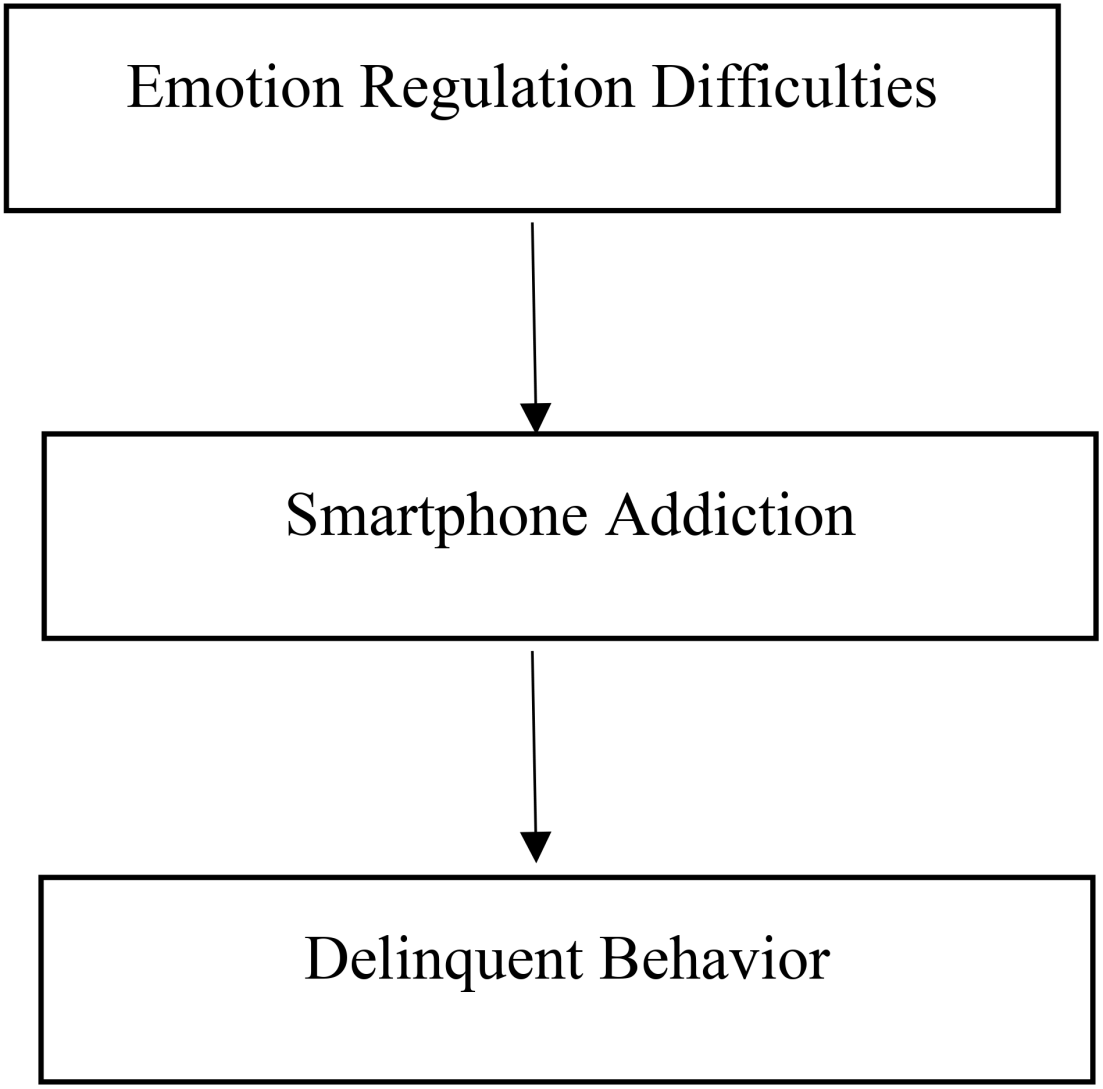
Conceptual model.

By integrating these theoretical perspectives, this study addresses the research gap in the Arab and Islamic contexts, where empirical investigations of the relationships among emotion regulation difficulties, smartphone addiction, and juvenile delinquency remain scarce. The study aims to clarify how emotional and behavioral vulnerabilities interact with digital technology to influence delinquent outcomes among male adolescents in institutional settings (See **Figure 1**).

Research questions

1. Is there a statistically significant relationship between smartphone addiction and delinquent behavior among juveniles?
2. Is there a statistically significant relationship between smartphone addiction and emotion regulation difficulties among juveniles?
3. Is there a statistically significant relationship between emotion regulation difficulties and delinquent behavior among juveniles?
4. Does smartphone addiction mediate the relationship between emotion regulation difficulties and delinquent behavior among juveniles?

## Materials and methods

### Participant and data collection

Data were collected through a survey that included sociodemographic characteristics, the Smartphone Addiction Proneness Scale (SAPS), the Difficulties in Emotion Regulation Scale (DERS), and the Self-Report Delinquency Scale (SRDS). The survey was distributed to 322 sentenced male juveniles aged 14 to 17, convicted of theft, alcohol or drug use, or property vandalism. Participants were recruited through convenience sampling from Juvenile Rehabilitation and Welfare Centers affiliated with the Ministry of Social Development in the governorates of Amman and Zarqa. Among the initial sample, 43.3% were aged 16–17 and attending high school. Regarding social media use via smartphones, 26.7% reported using Facebook, 6.7% Instagram, and 16.7% YouTube, while 59% used two or more of these platforms.

Of the 322 distributed questionnaires, 112 were excluded from the final analysis: 68 (21.1%) due to missing or incomplete data exceeding 20% of items, and 44 (13.7%) due to responses deemed invalid or inconsistent, indicating a lack of engagement. Listwise deletion was used to handle missing data, and Little’s MCAR test indicated that data were missing completely at random (p >.05), supporting the appropriateness of this approach. Sensitivity analyses comparing excluded and retained participants on key demographics (age, education level, and governorate) revealed no significant differences, minimizing concerns about potential selection bias.

A *post hoc* power analysis using G*Power confirmed that the final sample of 210 participants provided adequate statistical power (>0.80) to detect medium effect sizes in the proposed SEM/mediation model, supporting the sufficiency of the sample for the planned analyses.

The study focused exclusively on male sentenced juveniles, as the number of female juveniles in correctional facilities in Jordan is extremely limited, and their short duration of stay made their inclusion unfeasible. Consequently, the findings are representative only of institutionalized male juveniles within these rehabilitation centers and should not be generalized to the broader adolescent population.

The survey was administered in person between October and December 2024, with an average completion time of 10–20 minutes. Participants were briefed on the study’s objectives and assured that their responses would remain confidential and used solely for scientific research purposes. Ethical approval for the study was obtained from the Scientific Committee at [Blinded for review]. Consent for participation was granted by the Juvenile Rehabilitation and Welfare Centers, which have legal and ethical responsibility for the juveniles under institutional care. In addition, all participants provided verbal assent prior to participation. This procedure complies with the principles outlined in the Declaration of Helsinki and the Belmont Report, which permit verbal consent in cases where obtaining written consent is impractical or could compromise participant privacy. These measures ensured that participants’ rights and well-being were protected throughout the study.

### Measures

1. The Smartphone Addiction Proneness Scale (SAPS) is a tool designed to assess individuals’ tendency or proneness to smartphone addiction. Developed by Kim et al. (35), the SAPS consists of 29 items, distributed across several subscales: Disturbance of Adaptive Functions (9 items), Virtual Life Orientation (6 items), Withdrawal (7 items), and Tolerance (7 items). Each item is rated on a 5-point Likert scale, ranging from 1 (Strongly Disagree) to 5 (Strongly Agree). The SAPS has been previously applied in Jordanian adolescent samples (36), and the Arabic version was reviewed and adapted to ensure clarity and comprehensibility for participants
2. The Difficulties in Emotion Regulation Scale (DERS) is a tool designed to assess issues related to emotion regulation. Developed by Gratz & Roemer (37), this 36-item self-report scale asks respondents about their emotional responses, generating scores on several subscales: Nonacceptance of Emotional Responses (6 items), Difficulty Engaging in Goal-Directed Behavior (5 items), Impulse Control Difficulties (6 items), Lack of Emotional Awareness (6 items), Limited Access to Emotion Regulation Strategies (8 items), and Lack of Emotional Clarity (5 items). Each item is rated using a 5-point Likert scale, ranging from 1 (Almost Never) to 5 (Almost Always). DERS has been previously used in various Jordanian adolescent samples, and its Arabic version was carefully reviewed to ensure participants could understand all items.
3. The Self-Report Delinquency Scale (SRDS), developed by Elliott et al. (38), consists of 23 items drawn from the National Youth Survey (38). This scale assesses the frequency and severity of delinquent acts committed by adolescents, with items rated on a 5-point Likert scale, ranging from 1 (Never) to 9 (Two–Three times a day). The SRDS was previously applied in a pilot experimental study on a sample of male juveniles in Jordan by second author, confirming its cultural and contextual suitability. The Arabic version was reviewed to ensure clarity and comprehension for participants.

All instruments used in this study were translated into Arabic and culturally adapted to the Jordanian context. The results of reliability and internal consistency tests are reported in the Results section, confirming the appropriateness of these measures for the current sample.

### Data analysis

The collected data were analyzed using IBM SPSS Version 25, and path analysis was conducted using Structural Equation Modeling (SEM) in IBM AMOS Version 25. Prior to the main analyses, Confirmatory Factor Analyses (CFA) were performed separately for the Smartphone Addiction Proneness Scale (SAPS), the Difficulties in Emotion Regulation Scale (DERS), and the Self-Report Delinquency Scale (SRDS) to verify their construct validity in the current sample. All models demonstrated acceptable fit indices (CFI = .93–.96, TLI = .91–.95, RMSEA = .05–.07, SRMR = .04–.06), and all item loadings exceeded.50, confirming factorial validity. Internal consistency was assessed using Cronbach’s α and McDonald’s ω, both of which indicated satisfactory reliability across all subscales (α = .84–.89; ω = .85–.90). Item-total correlations ranged from.42 to.76, further supporting internal consistency. Descriptive statistics were computed for demographic variables. Although the Kolmogorov–Smirnov test was conducted to assess normality, SEM is robust to minor deviations from normality in large samples with ordinal Likert data. Skewness and kurtosis values for all variables were within acceptable ranges (± 2), confirming the suitability of the data for SEM analysis.

Before the main analyses, data screening procedures were performed to examine missing values and data completeness. Of the 322 distributed questionnaires, 112 were excluded from the final analysis: 68 (21.1%) due to missing or incomplete data exceeding 20% of items, and 44 (13.7%) due to inconsistent or invalid response patterns indicating lack of engagement. The final valid sample therefore comprised 210 participants (65.2% of the original sample). Missing data were evaluated using Little’s MCAR test, which indicated that data were missing completely at random (p >.05). Consequently, listwise deletion was applied. Sensitivity analyses comparing excluded and retained participants on key demographic variables (age, education level, and governorate) revealed no significant differences, minimizing concerns about selection bias.

In the SEM model, Emotion Regulation Difficulties, Smartphone Addiction, and Juvenile Delinquency were modeled as latent constructs, indicated by their respective subscales/items. Maximum Likelihood estimation was used to estimate the model parameters. Pearson’s correlation coefficients were computed to examine relationships among the study variables. Potential multicollinearity was assessed using Variance Inflation Factor (VIF) and tolerance values, all of which indicated no multicollinearity concerns. Model fit was evaluated using Chi²/df (CMIN/DF), Goodness-of-Fit Index (GFI), and Root Mean Square Error of Approximation (RMSEA).

To assess potential common-method bias arising from self-report measures, Harman’s single-factor test was conducted. Results indicated that a single factor did not account for the majority of variance (less than 40%), suggesting that common-method bias is unlikely to significantly affect the study results.

To test the significance of the indirect (mediated) effect of emotion regulation difficulties on juvenile delinquency via smartphone addiction, bootstrapping with 5,000 resamples was performed, and 95% confidence intervals were estimated. This approach ensures a robust assessment of both direct and indirect effects within the mediation model.

## Results

Our measures demonstrated excellent internal consistency between items, as indicated by the Cronbach’s alpha coefficient (α): the Smartphone Addiction scale (α = 0.88), the Emotion Regulation Difficulties scale (α = 0.89), and the Delinquency scale (α = 0.84). The results of the Kolmogorov–Smirnov test confirmed that the factors were normally distributed, with all p-values greater than 0.05. The arithmetic means and standard deviations suggest that male juveniles tend to report a high level of addiction to smartphones. With a relatively low standard deviation of 0.70, this indicates that, while most juveniles report high smartphone addiction levels. Subscales such as *Disturbance of Adaptive Functions* (M = 4.60) and *Virtual Life Orientation* (M = 4.40) also show high values, suggesting that they face significant disruptions in their daily functions due to smartphone use and demonstrates a strong orientation toward virtual life. The *Withdrawal* subscale, with a mean of 4.50, suggests that juveniles often experience withdrawal symptoms when not using their smartphones. Regarding emotion regulation difficulties, the overall mean of 4.60 reflects a high level of difficulty in regulating emotions, indicating that juveniles struggle significantly with emotional regulation. The subscales within this category also show high values, such as *Non-acceptance of Emotional Responses* (M = 4.70), which suggests that juveniles often find it difficult to accept their emotional reactions. The *Difficulty Engaging in Goal-directed Behavior* subscale, with a mean of 4.50, points to challenges in focusing on goals due to emotional disturbances. Other subscales, such as *Impulse Control Difficulties* (M = 4.60) and *Limited Access to Emotion Regulation Strategies* (M = 4.65), suggest that juveniles face difficulties controlling their impulses and have limited strategies for regulation their emotions. In contrast, the mean for delinquency was 4.20, indicating that juvenile delinquency is moderate, with juveniles reporting a relatively high frequency of delinquent behaviors.

As shown in **Table 1**, the Pearson correlation analysis reveals significant and positive associations between smartphone addiction and delinquency, as well as between emotion regulation difficulties and delinquency. Strong positive correlations were observed between the subscales of Smartphone Addiction (Disturbance of Adaptive Functions, Withdrawal, Tolerance) and Delinquency. For example, disturbance of adaptive functions and delinquency were strongly correlated (r = 0.63), indicating that higher levels of disturbances in daily functioning associated with smartphone use are related to higher levels of delinquent behaviors.

**Table 1:** Correlation analysis of smartphone addiction and delinquency, and emotion regulation difficulties among Male Juveniles.

Variable	Disturbance of adaptive functions	Virtual life orientation	Withdrawal	Tolerance	Non-acceptance of emotional responses	Difficulty engaging in goal-directed behavior	Impulse control difficulties	Lack of emotional awareness	Limited access to emotion regulation strategies	Lack of emotional clarity	Delinquency
Disturbance of adaptive functions	1.00	0.56**	0.43**	0.36*	0.65**	0.47**	0.53**	0.40*	0.51**	0.59**	0.63**
Virtual Life Orientation	0.56**	1.00	0.50**	0.45**	0.48**	0.38*	0.42*	0.30*	0.47**	0.50**	0.52**
Withdrawal	0.43**	0.50**	1.00	0.55**	0.41*	0.32*	0.45**	0.35*	0.41*	0.43**	0.51**
Tolerance	0.36*	0.45**	0.55**	1.00	0.38*	0.35*	0.49**	0.31*	0.49**	0.46**	0.55**
Non-acceptance of Emotional Responses	0.65**	0.48**	0.41*	0.38*	1.00	0.60**	0.62**	0.58**	0.55**	0.64**	0.58**
Difficulty Engaging in Goal-Directed Behavior	0.47**	0.38*	0.32*	0.35*	0.60**	1.00	0.55**	0.50**	0.58**	0.55**	0.57**
Impulse Control Difficulties	0.53**	0.42*	0.45**	0.49**	0.62**	0.55**	1.00	0.65**	0.63**	0.60**	0.62**
Lack of Emotional Awareness	0.40*	0.30*	0.35*	0.31*	0.58**	0.50**	0.65**	1.00	0.52**	0.59**	0.61**
Limited Access to Emotion Regulation Strategies	0.51**	0.47**	0.41*	0.49**	0.55**	0.58**	0.63**	0.52**	1.00	0.67**	0.59**
Lack of Emotional Clarity	0.59**	0.50**	0.43**	0.46**	0.64**	0.55**	0.60**	0.59**	0.67**	1.00	0.65**
Delinquency	0.63**	0.52**	0.51**	0.55**	0.58**	0.57**	0.62**	0.61**	0.59**	0.65**	1.00

**Correlation is significant at the p < 0.01 level (2-tailed); *Correlation is significant at the p < 0.05

Similarly, emotion regulation difficulties, such as impulse control difficulties, non-acceptance of emotional responses, and lack of emotional clarity, were positively associated with delinquency, suggesting that juveniles with greater emotion regulation challenges tend to exhibit higher delinquency levels. Furthermore, smartphone addiction, particularly disturbance of adaptive functions (r = 0.65), was positively associated with emotion regulation difficulties, indicating that juveniles reporting higher difficulties in regulating their emotions also tend to report higher levels of smartphone addiction.

These results demonstrate associations among smartphone addiction, emotion regulation difficulties, and delinquency, without implying causal relationships, in line with the cross-sectional nature of the study.

**Table 2** presents the results of the path analysis, illustrating the relationships among Emotion Regulation Difficulties, Smartphone Addiction, and Juvenile Delinquency, assessed through Structural Equation Modeling (SEM). SEM is a robust multivariate technique that combines factor and path analyses to examine both direct and indirect relationships among latent constructs.

**Table 2:** Path analysis results using SEM.

Path	β (Standardized coefficient)	SE	CR	P	R² (Dependent variable)
Emotion Regulation Difficulties → Smartphone Addiction	0.45	0.08	5.625	0.000**	0.42
Smartphone Addiction → Juvenile Delinquency	0.32	0.09	3.555	0.001**	0.36
Emotion Regulation Difficulties → Juvenile Delinquency (Direct Effect)	0.25	0.10	2.500	0.012*	—
Emotion Regulation Difficulties → Juvenile Delinquency (Indirect via Smartphone Addiction)	0.14	0.05	2.800	0.005**	—

Standardized regression coefficients (β) and R² for dependent variables are reported. Model fit indices: χ² = 45.00, CMIN/DF = 2.22, GFI = 0.95, RMSEA = 0.035.A single asterisk (*) typically means that the result is statistically significant at p ≤ 0.05.The double asterisks (“**”) next to the p-values indicate statistical significance at the p ≤ 0.01 level.

The results indicate that smartphone addiction partially mediates the association between emotion regulation difficulties and juvenile delinquency. Specifically, adolescents with greater difficulties in regulating their emotions tend to report higher levels of smartphone addiction, which, in turn, is associated with increased delinquent behaviors. Furthermore, emotion regulation difficulties exert a significant direct effect on juvenile delinquency beyond the indirect pathway via smartphone addiction.

The model accounted for 42% of the variance in smartphone addiction (R² = 0.42) and 36% of the variance in juvenile delinquency (R² = 0.36), indicating moderate explanatory power. Model fit indices demonstrated an adequate fit: χ² = 45.00, CMIN/DF = 2.22, GFI = 0.95, RMSEA = 0.035. To assess the mediation effect, bootstrapping with 5,000 resamples was performed, yielding a 95% confidence interval for the indirect effect (CI = 0.06–0.23), confirming statistical significance.

Overall, these findings highlight meaningful direct and indirect pathways linking emotion regulation difficulties, smartphone addiction, and juvenile delinquency, providing valuable implications for preventive interventions. However, due to the cross-sectional and non-causal design of the study, the observed associations should be interpreted with caution.

## Discussion

To our knowledge, this study is one of the first to address emotion regulation difficulties among sentenced juvenile delinquents. The study’s objective was to contribute positively to the literature on juvenile delinquency by revealing its relationship to smartphone addiction and emotion regulation difficulties—a variable not previously investigated in the Arab and Islamic contexts. Furthermore, it aimed to explore the mediating role of smartphone addiction in the relationship between emotion regulation difficulties and juvenile delinquency.

Our results revealed that the juveniles exhibit high levels of smartphone addiction and report substantial difficulties in regulating their emotions, including problems with impulse control, non-acceptance of emotional responses, and a lack of emotional clarity. These emotional regulation challenges are associated with a higher frequency of delinquent behaviors. Furthermore, a significant positive relationship was found between smartphone addiction—which disrupts adaptive functioning—and delinquency.

These findings can be understood through the lens of General Strain Theory (GST), which posits that individuals who experience strain, such as failure to achieve valued goals or exposure to negative stimuli, may experience negative affective states including frustration, anger, or depression (30, 31). When adolescents lack adaptive emotion regulation strategies, they are more likely to engage in maladaptive coping behaviors, such as excessive smartphone use or delinquent acts, to alleviate strain. Strains perceived as unjust, high in magnitude, or associated with low social control are particularly likely to motivate engagement in delinquent behavior. In this context, smartphone addiction may serve as both a maladaptive coping mechanism and a facilitator of delinquency, providing immediate gratification and exposure to risk-laden content, consistent with GST predictions.

Similarly, Self-Regulation Theory explains that adolescents with insufficient self-regulatory capacity struggle to monitor, evaluate, and modify their behavior in response to internal and external cues (33, 34). Deficits in self-regulation increase vulnerability to impulsive smartphone use, emotional dysregulation, and engagement in antisocial behaviors, offering a mechanistic explanation for the observed associations. Juveniles who cannot implement adaptive coping strategies are prone to both emotional and behavioral dysregulation, supporting the observed links between emotion regulation difficulties, smartphone addiction, and delinquency.

Smartphone addiction is linked to juvenile delinquency, as excessive use can disrupt sleep, academic performance, and social interactions, potentially contributing to delinquent behavior. It also exposes adolescents to risky online behaviors such as cyberbullying and sexting, which may further relate to delinquency. Additionally, it may exacerbate emotional regulation difficulties, making impulsive and aggressive behaviors more likely (17, 39). From a theoretical standpoint, GST suggests that these risk exposures represent negative stimuli increasing strain, while Self-Regulation Theory emphasizes the adolescents’ reduced ability to monitor and control such behavior, explaining their susceptibility to antisocial outcomes.

Juveniles’ problematic smartphone use (PSU) is influenced by various psychological and social factors. Huang et al. (40) identified key contributors to PSU, including self-control ability, loss of control, parent-child relationships, and peer attitudes toward smartphone use. Bridge factors like peer pressure and the fear of missing out (FOMO) exacerbate smartphone addiction among adolescents. Constant smartphone accessibility allows engagement with media that may reinforce antisocial behaviors, potentially increasing delinquency. Kuss and Griffiths (17) emphasized that excessive smartphone use contributes to emotional dysregulation, creating a cycle of instant gratification that, when unmet, can lead to frustration and poor decision-making, associated with delinquent behavior. These mechanisms illustrate the interplay between strain, emotion regulation, and maladaptive coping described in GST and Self-Regulation Theory.

In Sudan, Adam (41) found a positive correlation between smartphone use and juvenile delinquency, suggesting that content laden with negative values can affect adolescents’ cognitive and emotional development, fostering delinquent behaviors. Emotional issues such as anxiety, stress, and low self-esteem may drive adolescents to seek relief through unhealthy coping mechanisms, including excessive smartphone use (19). Jeon (24) explored smartphone dependency and emotional problems as mediators between negative parenting and delinquency, finding that smartphone-dependent adolescents were more likely to exhibit delinquent behaviors under negative parenting. Participation in cultural activities weakened these associations, highlighting the protective role of social engagement in mitigating risks. Conversely, Alkabi and Mai (42) found strong correlations between academic failure and delinquency, noting that consumption of varied media content increased risk, but imitation of violent content had no direct effect, suggesting other factors like academic and social contexts play larger roles (8).

Building on these findings, consistent with recent evidence in primary school students, our results indicate that difficulties in emotion regulation associated with smartphone addiction may exacerbate negative behavioral outcomes. Specifically, adolescents with greater emotion regulation difficulties were more likely to report higher levels of smartphone addiction, which, in turn, was associated with increased delinquent behaviors. This aligns with prior research demonstrating that negative emotions and impaired emotional control can significantly affect interpersonal relationships and behavioral outcomes (16). While our study focused on institutionalized male juveniles rather than family dynamics, the differential impact of smartphone addiction dimensions on negative emotions underscores the importance of targeted interventions aimed at enhancing emotional regulation skills to reduce maladaptive behaviors.

The findings of the current study regarding the relationship between smartphone addiction and difficulties in emotion regulation align with recent research emphasizing the multidimensional nature of smartphone addiction. A study by Choi et al. (43) demonstrated that different facets of smartphone addiction are differentially associated with cognitive emotion regulation strategies among young adults, suggesting that certain addictive behaviors may exacerbate negative emotions while others could serve as coping mechanisms. This resonates with our results, which indicate that emotion regulation difficulties are significantly linked to higher smartphone addiction and, in turn, to increased juvenile delinquency. These converging findings underscore the importance of considering the nuanced patterns of smartphone use when designing interventions, highlighting that preventive and therapeutic approaches should target specific addiction dimensions and their impact on emotional regulation. Moreover, integrating emotion regulation strategies into intervention programs may be particularly beneficial in mitigating the maladaptive consequences of excessive smartphone use among adolescents.

Our findings on the relationship between smartphone addiction and emotional regulation difficulties align with recent evidence indicating that smartphone addiction is closely linked to problematic behaviors, including aggression. Deng et al. (44) demonstrated that smartphone addiction among adolescents can predict both internalized and externalized aggression, with depression mediating some of these associations. These results complement our study by highlighting that the emotional and behavioral consequences of smartphone addiction are multifaceted and may vary depending on underlying psychological mechanisms. While our study focused on delinquent behaviors rather than aggression per se, the evidence from Deng et al. (44) reinforces the notion that excessive smartphone use exacerbates difficulties in emotion regulation, which in turn can manifest as various maladaptive behaviors. Incorporating these insights allows for a deeper understanding of the pathways linking emotion regulation deficits, smartphone addiction, and externalizing behaviors, and underscores the importance of addressing emotional coping strategies in preventive interventions for institutionalized juveniles.

Our results further indicate that smartphone addiction partially mediates the relationship between emotion regulation difficulties and juvenile delinquency, while the direct effect of emotion regulation difficulties remains significant. Difficulties in emotion regulation are associated with higher smartphone addiction, which in turn is linked to delinquent behaviors. Other mediating mechanisms may include affiliation with deviant peers and exposure to peer delinquency, which amplify the impact of emotional deficits on delinquent behavior (45, 46). Negative affect and peer delinquency have been identified as mediators linking trauma to juvenile delinquency, demonstrating complex pathways from emotional difficulties to problem behaviors. Family environment and parenting behaviors are robust predictors, with low monitoring, high hostility, and psychological control explaining a significant portion of variance in delinquency (47). The integration of these findings with GST underscores how environmental strains interact with emotional and self-regulatory deficits to increase delinquency risk. Consistent with these findings, Al-Zawahreh (27) reported that sentenced male juveniles receive moderate social support, with a “significant person” (parent, supervisor, or close individual) and family being the most influential sources. Support from these figures was positively associated with problem-focused and emotional coping, whereas avoidant coping showed no significant relationship. These results complement our study by highlighting how close personal relationships can enhance adaptive coping strategies, which, when combined with emotion regulation difficulties and excessive smartphone use, may influence delinquent behavior. Integrating these insights reinforces the theoretical framework of General Strain Theory and Self-Regulation Theory in understanding the interplay between social, emotional, and digital factors in juvenile delinquency.

Emotion regulation difficulties also show direct associations with delinquency, independent of smartphone addiction. Deficits in recognizing, selecting, applying, and monitoring regulatory strategies (48) contribute to impulsivity, aggression, and poor decision-making, increasing delinquency risk (49, 50). Poor emotional regulation is linked to heightened antisocial behavior and disrupted peer relationships (51), as well as other behavioral risks. Early interventions to enhance emotional regulation are crucial to prevent delinquency (52). Emotional regulation mediates the relationship between verbal learning and psychopathology, with deficits increasing the likelihood of anxiety, depression, behavioral disorders, and academic failure, further escalating delinquency risk (53). These associations can be theoretically explained through GST, as inadequate coping with strain produces maladaptive outcomes, and Self-Regulation Theory, as failure to monitor and adjust emotions leads to antisocial actions.

The style of emotion regulation is closely linked to emotional expression and delinquency. Juveniles with poor emotional regulation may express emotions aggressively or impulsively, manifesting in criminal behavior (49, 54). Emotional regulation training in educational and rehabilitation programs may mitigate psychopathology and delinquency by fostering healthier emotional expression and social functioning (55).

Finally, problematic smartphone use interacts with emotional regulation difficulties, negative parenting, and social influences to increase delinquency risk. Adolescents struggling to regulate anger may respond impulsively to stress and conflict, leading to aggressive behaviors (49). Poor emotional regulation impairs decision-making and increases susceptibility to risky behaviors (50). Interventions enhancing emotional regulation, academic performance, and positive peer engagement can reduce delinquency risk, while self-compassion and psychological strategies act as protective factors (56). Addressing the combined influence of emotional regulation deficits, environmental factors, and modern technology allows a comprehensive understanding and mitigation of juvenile delinquency risks, consistent with GST and Self-Regulation Theory, and provides a theoretical rationale for the mechanisms observed in our study.

## Limitations and future research

This study has several limitations that should be acknowledged. First, its cross-sectional design limits the ability to draw causal conclusions, as the observed associations between emotion regulation difficulties, smartphone addiction, and juvenile delinquency reflect a single point in time. Consequently, alternative explanations, such as reverse causation or the influence of unmeasured third variables, may account for some of the reported relationships. Future research using longitudinal or experimental designs is recommended to clarify the directionality of effects and provide stronger evidence for causal mechanisms.

Second, the study relied on self-report measures, which may introduce biases, including social desirability, where participants could underreport delinquent behaviors or overestimate their emotional regulation capacities. Third, the correctional environment itself may have influenced responses due to psychological stress, continuous monitoring, or fear of repercussions, potentially affecting the accuracy of the data. Fourth, although this study focused on emotion regulation difficulties and smartphone addiction, other relevant factors—such as family support, exposure to domestic violence, or broader socio-cultural influences—were not directly measured and may also contribute to delinquent behaviors. Fifth, the use of translated instruments may introduce minor variations in meaning compared to the original measures, despite efforts to ensure reliability and validity.

Another key limitation relates to the generalizability of the findings. The study exclusively included male sentenced juveniles in Jordan, which limits the applicability of the results to female adolescents, community-based populations, or juveniles from other cultural or geographical contexts. The small number of female juveniles in correctional facilities, along with their short duration of stay, made it challenging to include them. Consequently, the experiences, emotion regulation patterns, and behavioral outcomes of female juveniles may differ from those of males. Future research should aim to include female participants, potentially through multi-site collaborations, longer-term longitudinal designs, or targeted recruitment strategies, to provide a more comprehensive understanding of emotion regulation difficulties, smartphone addiction, and delinquency across genders. Additionally, examining gender differences could offer insights into whether intervention strategies need to be gender-specific to effectively address emotional and behavioral dysregulation in juveniles.

Furthermore, the study focused solely on incarcerated juveniles, which may not reflect the experiences of adolescents in non-correctional settings. One potential avenue for future research is the inclusion of a non-delinquent control group. Comparing incarcerated juveniles with their non-delinquent peers would allow researchers to determine whether emotion regulation difficulties and smartphone addiction are uniquely elevated among delinquent youth or reflect broader developmental patterns. Such comparisons could strengthen causal inferences, clarify baseline differences, and inform targeted intervention programs for delinquent adolescents rather than applying general strategies to all youth populations.

Future research directions also include adopting a longitudinal approach to investigate the long-term effects of smartphone addiction and emotion regulation difficulties on juvenile delinquency. Longitudinal studies would help clarify causal relationships and examine changes over time between these factors. Expanding the sample to include adolescents from diverse socioeconomic and cultural backgrounds would enhance generalizability and allow prevention strategies to be tailored to different contexts. Additionally, future studies could explore moderating factors, such as age, gender, type of delinquent behavior, and urban versus rural residence, which may influence the strength or nature of mediation effects.

Moreover, examining the specific role of different social media platforms in shaping adolescent behavior could provide more insight into how digital environments contribute to delinquency. Exploring family dynamics, such as parenting styles and emotional climate, in relation to smartphone addiction and emotion regulation could lead to more effective family-based interventions. Finally, the lack of measurement of potential confounding variables, such as socioeconomic status, parental education, substance use, type of offense, and sentence length, represents another limitation. These factors were not the focus of the current research, and their absence may limit the generalizability of the findings, leaving room for unmeasured influences. Future studies are encouraged to include these variables to provide a more comprehensive understanding of factors contributing to emotion regulation difficulties, smartphone addiction, and delinquent behavior among juvenile offenders.

## Conclusion

In this study, which included 210 sentenced juvenile delinquents, the significant roles of both smartphone addiction and emotion regulation difficulties in juvenile delinquency were underscored. The findings highlight that smartphone addiction not only disrupts the daily functioning of adolescents but also exacerbates emotional regulation issues, which in turn increases the likelihood of engaging in delinquent behaviors. Moreover, the study identifies smartphone addiction as a key mediator in the relationship between emotion regulation difficulties and juvenile delinquency, suggesting that addressing these emotional challenges could reduce the risk of delinquency in adolescents. Interventions that focus on improving emotion regulation, managing smartphone use, and fostering healthier coping mechanisms could play a crucial role in preventing juvenile delinquency. Further research is needed to explore these relationships in greater depth and to develop more effective strategies for intervention and prevention.

## Data Availability

The raw data supporting the conclusions of this article will be made available by the authors, without undue reservation.
